# Deglutition-Induced Atrial Fibrillation in a Previously Healthy Adult Male: A Rare Trigger Unmasked After Medication Discontinuation

**DOI:** 10.7759/cureus.94395

**Published:** 2025-10-12

**Authors:** Adel Kenawi, Ayman El Masri, Hatem Abdelnasser

**Affiliations:** 1 Cardiology, Tameside General Hospital, Ashton-Under-Lyne, GBR; 2 Acute Medicine, Tameside General Hospital, Ashton-Under-Lyne, GBR

**Keywords:** adrenergic-driven atrial fibrillation, arrhythmia, deglutition-induced af, paroxysmal atrial fibrillation, postprandial atrial tachyarrhythmia, swallowing-induced af, swallowing-induced tachyarrhythmia, sympathetic overdrive atrial fibrillation

## Abstract

We report a rare case of swallowing-induced paroxysmal atrial fibrillation (AF) in a previously healthy 50-year-old male. Initially diagnosed with paroxysmal AF and successfully managed on flecainide and bisoprolol, the patient became asymptomatic, and his Holter monitor prior to the latest admission showed he maintained sinus rhythm. However, after discontinuation of antiarrhythmic therapy, he presented with recurrent palpitations. Continuous telemetry monitoring during readmission revealed episodes of AF triggered by swallowing liquids, resolving shortly thereafter. Investigations ruled out structural heart disease, thyroid dysfunction, and electrolyte abnormalities. This case highlights the importance of recognizing atypical triggers of AF.

## Introduction

Swallowing is uncommonly associated with cardiac arrhythmias [1]. Atrial fibrillation (AF) is the most common cardiac arrhythmia in adults. While common triggers include stress, alcohol, and electrolyte disturbances, mechanical or reflex triggers such as swallowing are rare and under-recognized [2]. Swallowing-induced atrial tachyarrhythmia is rare and accounts for 0.6% [3]. Swallowing-induced AF is thought to involve vagal or oesophageal-cardiac reflex pathways but remains poorly understood [1,2]. It is important to note down the history of presenting complaint, detailed history, and examination to highlight the relation between swallowing and the tachyarrhythmia, as well as excluding other aetiologies such as drugs, metabolic, ischaemic, or structural heart disease [2]. This case sheds light on this uncommon presentation.

## Case presentation

A 50-year-old previously healthy and physically active man presented to the Emergency Department complaining of recurrent palpitations. Six months previously, he had been admitted with paroxysmal AF and was initiated on flecainide 50 mg twice daily and bisoprolol. Echocardiogram at that time showed a normal left ventricular ejection fraction and no significant valvular abnormalities. He remained asymptomatic and in sinus rhythm for several months.

However, his medications were discontinued. Weeks later, he began experiencing recurrent palpitations and presented again to the ED. He was found to be in AF with a rapid ventricular response. His heart rate was 149 beats per minute. He was hemodynamically stable with an unremarkable physical examination, except for an irregular pulse on cardiac auscultation.

**Figure 1:**
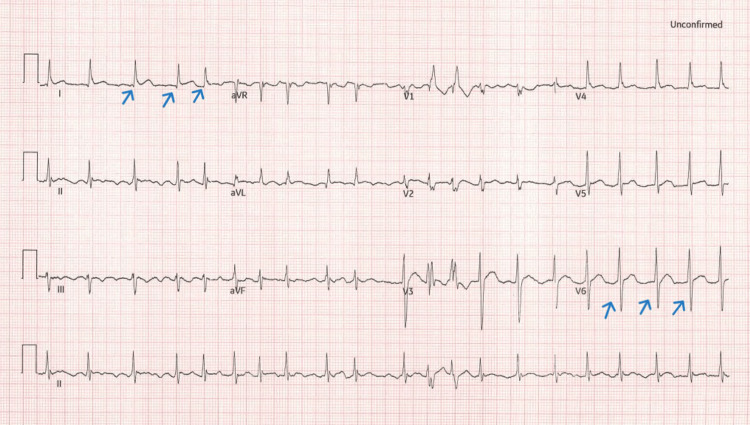
ECG shows atrial fibrillation with a ventricular response (blue arrows) rate about 120 beats per minute

**Figure 2:**
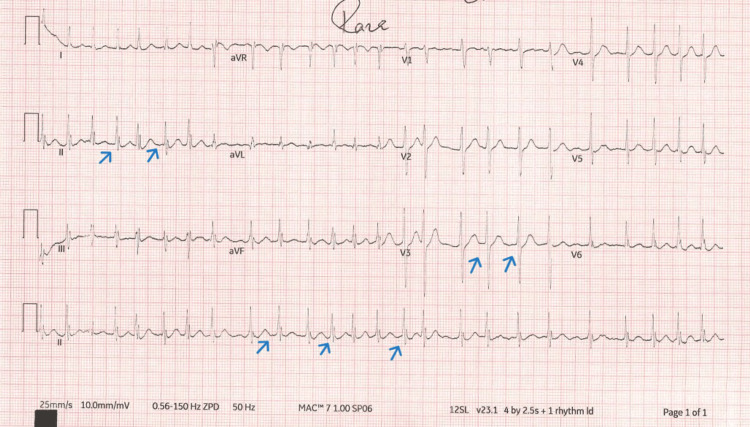
ECG shows atrial fibrillation with a rapid ventricular response rate (blue arrows) and heart rate about 145 beats per minute

**Figure 3:**
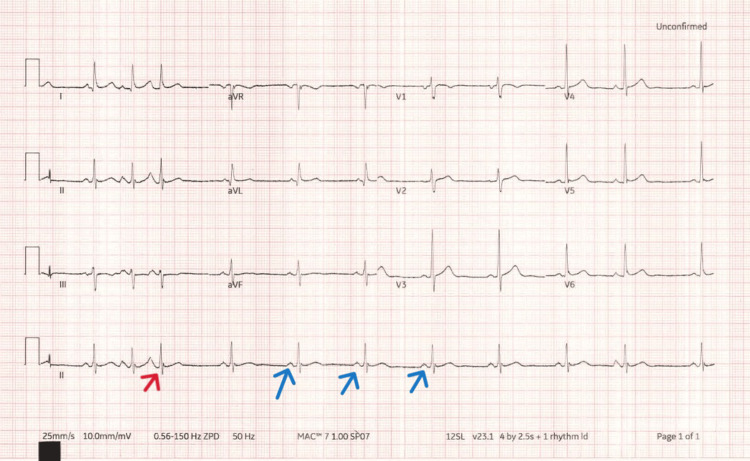
ECG shows the presence of P-wave before QRS complex (blue arrows), indicating sinus rhythm There was one premature atrial complex (red arrow).

The patient underwent ECG, transthoracic echocardiography, routine blood tests, and continuous telemetry monitoring. ECG initially confirmed AF with rapid ventricular response (Figures 1-2). He was given bisoprolol 3.75 mg twice daily, and subsequent ECG showed that he was back to sinus rhythm (Figure 3).

**Figure 4:**
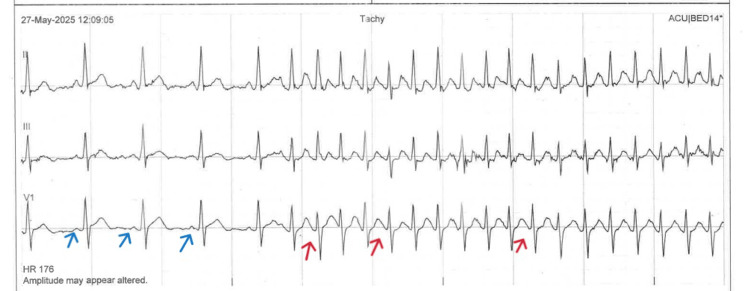
Telemetry rhythm strip showing sinus rhythm initially (blue arrows) and then AF with rapid ventricular response while drinking water (red arrows)

**Figure 5:**
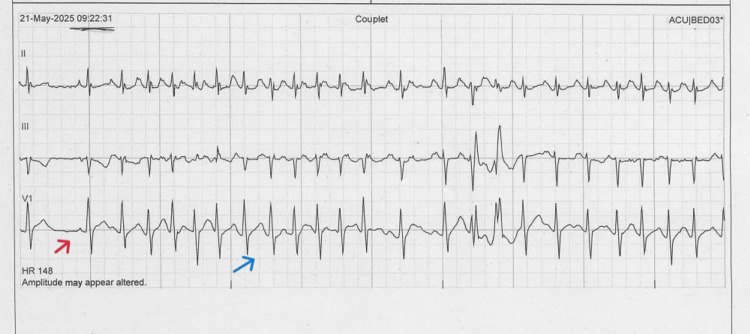
Telemetry rhythm strip showing sinus rhythm (red arrows) and AF with rapid ventricular response while drinking water (blue arrows)

His repeat echocardiogram showed normal bi-ventricular systolic function, an estimated ejection fraction of ~61%, normal LV diastolic function, no significant valvular abnormalities, low probability of pulmonary hypertension, dilated right atrium, and borderline dilated left atrium. Laboratory tests, including full blood count, electrolytes, and renal, liver, and thyroid function, were all normal. Telemetry monitoring showed frequent episodes of AF, temporally associated with swallowing liquids (Figures 4-5).

The patient was admitted for cardiac monitoring and medical optimisation. Notably, telemetry revealed that AF episodes were consistently triggered by the act of swallowing liquids and terminated spontaneously shortly after. Given this pattern, a diagnosis of swallowing-induced AF was considered. Rate control was maintained, and options for rhythm control and longer-term management were discussed with the patient.

The patient was restarted on flecainide and bisoprolol, and he was discharged with outpatient cardiology follow-up. A six-day ambulatory ECG monitor was arranged as an outpatient to evaluate the effectiveness of pharmacological therapy in controlling and preventing the occurrence of this phenomenon. He was advised to avoid cold or carbonated beverages and to observe for symptom recurrence.

## Discussion

Deglutition-induced arrhythmia is a rare phenomenon, representing less than 1% of reported AF cases [1]. Swallowing-induced dysrhythmias are divided into two categories: bradyarrhythmias and tachyarrhythmias, the former being more common than the latter [1]. Tachyarrhythmia during swallowing was first described by Sakai and Mori in 1926 under the name of ‘’Schlucktachycardie’’ [2]. Most cases occur in adult men and recur frequently within a short time while swallowing [3]. Most cases of pre-syncope and syncope with swallowing are associated with bradyarrhythmias and are often associated with underlying structural abnormalities of the heart (i.e., ischaemic heart disease), gastrointestinal system (i.e., diffuse oesophageal spasm, oesophageal diverticulum, achalasia) or both [4].

Deglutition-induced AF is an unusual form of AF likely mediated by vagal stimulation or oesophageal-cardiac neural reflexes. It may occur in structurally normal hearts and is often underdiagnosed unless specifically monitored during provocative manoeuvres [4]. In this case, the relationship between swallowing and AF onset was evident and reproducible.

The mechanism by which deglutition-induced arrhythmia remains ambiguous. It is suggested that the compression against the left atrium by a dilated oesophagus induces the arrhythmia. Furthermore, the process might be induced by either wet or dry swallow, with a significant difference in its burden in favour of a more consistent relation to wet swallow, resulting in vagal stimulation and vasovagal reflux-induced tachyarrhythmia. Another astonishing finding was the difference in the lower oesophageal sphincter pressure between the wet and dry swallow, where it was much higher in the wet swallow episode [1]. Another proposed mechanism involves activation of the efferent and afferent branches of the vagus nerve from increased intra-oesophageal pressure. As the afferent and efferent vagal reflexes are activated with primary peristalsis from swallowing or oesophageal dilation, the stimulation can shorten the refractory period, resulting in a nonuniform excitation of the atrial myocardium and atrial tachycardia. Finally, a final hypothesis involves activation of the sympathetic nervous system, which could result in nonuniform atrial depolarization and trigger AF [1].

In one published case report, the causative agent was salbutamol, which suggests that adrenergic stimulation plays a role in driving this pathology [2]. Another case report about a 51-year-old with a history of bypass tract of left posterior septal ablation for atrioventricular re-entrant tachycardia (AVRT) [3]. This patient presented again with swallowing-induced atrial tachycardia for which different modalities of pharmacological testing were assessed. Isoproterenol intravenous infusion caused tachycardia captured on cardiac monitor, similar to that observed during swallowing, indicating sympathetic nerve activation [3]. There is also a report of paroxysmal AF that was triggered by the ingestion of ice-cold water following physical exercise [5].

In terms of symptoms, palpitations, which were rarely brought on by burping, were the most common presenting symptom, followed by syncope. Some patients reported lightheadedness consistently associated with eating [6-9].

Management may include antiarrhythmic drug therapy (AADs), avoidance of triggers, and, in some cases, catheter ablation of the arrhythmogenic focus [1,2]. Beta-blockers alone are observed to be ineffective in treating this condition alone and the addition of class 1c antiarrhythmic drugs such as propafenone and flecainide was proven successful in managing this category of patients [2]. In patients without structural heart disease, flecainide has been demonstrated to be safe and well-tolerated relative to other AADs. Chronic administration of oral flecainide has a class IA recommendation in current guidelines from the United States and the EU for the suppression of AF in patients without structural heart disease with recurrent paroxysmal or persistent AF [10]. Flecainide has proven to be more effective than other AADs for the acute termination of recent onset AF [11]. Radiofrequency catheter ablation is an option in pharmacological treatment-refractory cases, which is successful in 100% of cases [2]. Radiofrequency catheter ablation is an important modality of treatment in patients with poor drug compliance, epileptic disorders, or those who prefer to live a drug-free life [4]. There are reported surgical procedures to alleviate this burden by intrapleural repositioning of the oesophagus [3] and circular oesophageal myotomy [2,10]. It was reported in one study that radiofrequency catheter ablation of the arrhythmogenic source has also been attempted successfully on swallowing-induced tachyarrhythmias refractory to medical treatment [12].

## Conclusions

Deglutition-induced AF is a rare and often under-recognised condition triggered by swallowing, likely involving complex interactions between vagal and sympathetic nervous system pathways, as well as mechanical factors related to the oesophagus. Palpitations were the most common presenting symptom, followed by syncope. Variable potential associations were reported.

Treatment options include pharmacological therapy; particularly, class 1C antiarrhythmic agents have shown effectiveness. Radiofrequency catheter ablation of the arrhythmogenic source can be tried in cases refractory to medical treatment. Understanding the underlying mechanisms and individual patient triggers is essential for tailored therapy, and in select cases, surgical interventions may provide additional relief. Early recognition and appropriate treatment can significantly improve outcomes in affected patients.
